# Performance comparison of linear and non-linear feature selection methods for the analysis of large survey datasets

**DOI:** 10.1371/journal.pone.0213584

**Published:** 2019-03-21

**Authors:** Olga Krakovska, Gregory Christie, Andrew Sixsmith, Martin Ester, Sylvain Moreno

**Affiliations:** 1 Digital Health Hub, Simon Fraser University, Surrey, British Columbia, Canada; 2 Science and Technology for Aging Research Institute, Simon Fraser University, Surrey, British Columbia, Canada; 3 Department of Gerontology, Simon Fraser University, Vancouver, British Columbia, Canada; 4 Department of Computer Science, Simon Fraser University, Vancouver, British Columbia, Canada; 5 School of Interactive Arts and Technology, Simon Fraser University, Surrey, British Columbia, Canada; University College London Hospitals NHS Foundation Trust, UNITED KINGDOM

## Abstract

Large survey databases for aging-related analysis are often examined to discover key factors that affect a dependent variable of interest. Typically, this analysis is performed with methods assuming linear dependencies between variables. Such assumptions however do not hold in many cases, wherein data are linked by way of non-linear dependencies. This in turn requires applications of analytic methods, which are more accurate in identifying potentially non-linear dependencies. Here, we objectively compared the feature selection performance of several frequently-used linear selection methods and three non-linear selection methods in the context of large survey data. These methods were assessed using both synthetic and real-world datasets, wherein relationships between the features and dependent variables were known in advance. In contrast to linear methods, we found that the non-linear methods offered better overall feature selection performance than linear methods in all usage conditions. Moreover, the performance of the non-linear methods was more stable, being unaffected by the inclusion or exclusion of variables from the datasets. These properties make non-linear feature selection methods a potentially preferable tool for both hypothesis-driven and exploratory analyses for aging-related datasets.

## Introduction

Within the field of statistical gerontology, there has been increasing use of large databases to explore relationships between key factorsand some outcome variable(s) of interest (dependent variable(s)). Indeed, several survey initiatives have been set up to track the biological, social and lifestyle factors that affect health and quality of life throughout the lifespan, i.e.Health and Retirement Study [1], Wisconsin Longitudinal Study[2] Canadian Longitudinal Study on Aging [3], National Population Health Survey [4]These databanks are a valuable resource that can be used to identify and quantify the factors affecting health in aging. In turn, the results of these analyses can empower key stakeholders, including end users and policy makers, to make informed decisions for themselves and optimized decisions at higher levels, i.e. at the level of healthcare systems.

However, the use of these datasets presents some significant challenges if they are to be used optimally to provide us with convincing results, strong evidence, and useful information. For example, it is important that researchers identify and only use variables within a database that are relevant to the outcome in question. Typically, quantitativeanalysiswithin gerontology has used linear methods (methods, that assume relationships)as a means of simplifying data and identifying relevant variables[5–8].However, the use of linear methods in databases where there are non-linear relationships can yield to misleading results[9]. A systematic review of 893 papers illustrated, that 92% of incorporated papers using linear methods were unclear about assumptions of the methods used [10].The purpose of this paper is to provide a systematic evaluation of different approaches to feature selection.We will do this firstly by reviewing and discussingin more detail some of the key problems and limitations in the analysis of large survey databases, including variable selection when dealing with non-linear relationships. Secondly, we quantitatively compare a range of different linear and non-linear methods (by non-linear methods we imply methods, that do not necessarily assume linear relationships) in order to evaluate their relative performance in terms of selecting relevant features from two example large survey databases.

## Background

It is not uncommon for large survey databases to store dozens or hundreds of different measurements for each person (we refer to these measurements herein as *features*). Given their size and complexity, it is not usually practical for researchers to assess how all factors within a database interact to determine an outcome of interest (say, mortality rate). Instead, researchers will often select a handful of features and assess the predictive ability of these features using a variant of regression such as linear regression. Unfortunately, both of these operations—feature selection and prediction—are potentially problematic for the analysis of many large survey databases. Here, we outline two major issues inherent to this analytic technique and offer an alternative approach, which may be better suited for the analysis of data within these survey databases, when it is reasonable to assume non-linear relationships.

The first major issue pertains to the process of correctly identifying relevant features from irrelevant ones. In nearly all aging-related datasets analyses, experimenters must identify and select features that are relevant to the dependent variables of interest and reject all other, irrelevant features. Broadly construed, this is typically done using one of two, non-exclusive approaches. The first is to select features based on prior knowledge and one or more a priori hypotheses. We refer to this as model selection. For example, a researcher may be curious about the effects of alcohol consumption on mortality rates. The researcher could then select features that are relevant to the question of interest (e.g. number of alcoholic units consumed per week), along with other features that they believe may confound the results (e.g. education level), and ignore all other, presumably irrelevant features.

Although this practice is employed frequently (more than 50% of papers that analyzed “life activities” in HRS [1]dataset in 2012–2017 used linear methods), it is potentially problematic for several reasons. Obviously, the predictive accuracy of a solution is only as good as the features selected to model it, and model selection can fail when relevant features are not selected for inclusion, irrelevant features are selected for inclusion, or both. Although prior knowledge can help guide this manual process, there is no guarantee that this knowledge will lead to the selection of all relevant features and the rejection of all irrelevant ones. In fact, as the number of features in a database increases, the likelihood of erroneous model selection approaches certainty, a problem referred to as the model problem. Model selection is also impractical for exploratory data analyses, in which researchers have weak (or no) a priori hypotheses or knowledge to guide selection. Finally, model selection is likely insufficient to eliminate the problem of multiple collinearity, which occurs when one or more features are correlated with other features.

Rather than selecting features manually, researchers can also use statistical approaches that transform the original, higher-dimensional feature space into a lower-dimensional space. For example, exploratory factor analysis and principal components analysis[11, 12], explain patterns of inter-correlated data by way of a small number of underlying factors. Compared to manual feature selection, these factor analyses are agnostic to a priori hypotheses and are therefore more appropriate for exploratory data analyses. Moreover, because they are data-driven, they also minimize collinearity (and maximize parsimony) by explaining the greatest amount of variance in the original data with the fewest number of underlying factors. However, because these are transformational approaches, the computed factors represent a combination of the underlying, original features. In other words, a computed factor does not represent any one original feature in the dataset, but rather a complex combination of all features in the dataset. As a consequence, the interpretation of these results can be difficult and somewhat subjective.

Building atop this, a second major issue pertains to the process of simplifying datasets that contain non-linear relationships between variables. It is thought that many non-linear relationships exist linking variables within the health sciences[13–15]. These non-linear dependencies further exacerbate the challenge of correctly reducing the dimensionality of a dataset, as many linear methods can fail to adequately identify them. The performance of linear methods is also negatively impacted if datasets include extreme values and skewed distributions, both of which are, again, common in survey datasets.

Linear methods have been widely adopted for problems in data projection and dimensionality reduction. They still remain the first choice in the context of gerontology, but without being optimal. Here, we evaluate the performance of the most frequently used linear methods as well as non-linear methods on survey datasets.

In applications to large survey datasets, identification of the relevant features is usually done by *automatic feature selection*. Automatic feature selection derives a simplified model from the statistical properties of the underlying data, only this time by selecting the original features in the dataset. This process, while powerful, comes at a combinatorial cost. A brute-force solution—that is, one that finds the best solution by systematically assessing all possible combinations of underlying features—is computationally unfeasible for large survey databanks, which can easily contain hundreds or thousands of features. Instead, an approximate solution must be estimated, typically using one of three broad categories of selection methods: *filter*, *wrapper*, *and embedded*[16, 17]. These methods differ in how they select relevant features from irrelevant ones and thus merit a brief introduction.

Filter methods are a pre-processing step that scores each feature using a statistical measure (e.g. correlation coefficient), ranks all features on this measure, and rejects features that fall below a cut-off criterion. Filter methods are by far the least computationally demanding method, because they operate on each feature individually and ignore dependencies between them. However, this same approach means that filter methods do not solve the problem of multicollinearity, which can in turn lead to relatively poor performance relative to other techniques. In recognition of this, one of the common application of the filter methods is identifying relevant features for future modelling.

Wrapper methods are a category of approaches in which features are selected and assessed, in conjunction with other features, in their ability to account for the variance in the underlying data. An algorithm iteratively learns to select the combination of features that best explains the data. Common approaches to doing this include forward and backwards selection approaches, in which the algorithm starts initially with either none or all of the variables (respectively), and adds/removes variables until the model no longer improves. This approach is vastly employed in both linear and non-linear methods. Given its iterative nature, wrapper methods are relatively expensive computationally, and the typical forward/backward selection methods have both been shown to be potentially experimentally problematic in terms of identifying most relevant subset of features[18, 19]. There is also a risk that wrapper methods can overfit the data, meaning that the solution accounts for random noise and in actuality has relatively poor predictive performance when applied to new data on which it has not been trained.

Lastly, embedded methods are similar to wrapper methods in that an algorithm iteratively learns to select the features that best contribute to the accuracy of the overall solution. They include interactions between features in generating the model, which typically makes them superior to filter methods for prediction, and less likely to overfit the data than wrapper methods. Although these methods are beyond the scope of the present study, embedded approaches have shown promise in other recent studies that have focused on the analysis of large datasets with multiple variable interactions[20]

The goal of the present study was to quantitatively compare the performance of different linear (i.e., commonly studied in gerontology field) and nonlinear selection methods for the identification of relevant features within the two main survey databases (i.e., Wisconsin Longitudinal Study of Aging database, and Health Retirement Study) with applications to non-linear associations in data. To do this, we compared the performance of several linear methods (regression) widely used in gerontology versus non-linear (filter) feature selection methods using two main survey databases (WLS[2], and HRS[1]). Note here, that by "linear feature selection methods" we imply methods, that assume linear functional relationship between features and target variables, while "non-linear methods" do not have this interim assumption. In order to validate our results, we further tested those methods using synthetic datasets. Although we did not expect linear and non-linear methods to differ in their ability to identify linearly dependent features, we did hypothesize that non-linear methods would be superior at identifying non-linearly dependent features. As a result, non-linear based selection approaches may offer a more robust tool for feature identification, classification, prediction and machine learning applications for gerontology researchers.

## Methods

The performance of a given statistical method depends on the underlying data to be analyzed. Therefore, an important preliminary step is to understand the properties of the data before commencing any analysis[21]. Here, we are interested in the extraction of relevant features from large social science datasets, which consist primarily of questionnaires filled by respondents, their proxies or reviewers[22]. To make a questionnaire simpler for respondents, questions are routinely presented in multiple choice formats, which maps continuous variables into discrete categories, with the number of categories typically ranging between three to seven. Respondents are occasionally asked to provide an exact number to a given question, and as a result the risk of erroneously splitting a response into categories is believed to be relatively high. For example, a respondent performing an activity five times per week may either report it as “daily” or “several times a week”.

Given this, it was important to understand how the various feature selection approaches (see next section) performed under these analysis conditions. Important parameters here include the level of noise obscuring the relationship between variables, the number of samples available for analysis, and the effects of discrete versus continuous variable representations. We therefore assessed performance in two ways. First, we constructed a series of synthetic datasets that mimic the noisy and non-linear nature of many survey datasets. Because the associations between variables were known in advance, we would be able to quantitatively gauge the performance of the different selection methods in identifying relevant features and discarding irrelevant ones (see ‘Synthetic Data’, below). Second, we further gauged the performance of the different selection methods using two representative datasets, the Wisconsin Longitudinal Study[23], and Health and Retirement Study[1]. Here, we relied on a priori knowledge to assess each method’s ability to identify previously-established dependencies between the variables within the dataset—namely, the effect of certain lifestyle activities on overall health (see ‘Representative Data’, below).

For both the synthetic and representative datasets, each feature was identified as either important or unimportant by each feature selection method. For linear methods, we assumed that a selected feature was important if the corresponding coefficient was not equal to zero at a .05 significance level. For the filter methods, we assumed that a selected feature was important if the feature and target variable werenot independent at a .05 significance level. Finally, the performance of each selection method was computed using F_1_ scores, which represents the harmonic average of the precision and sensitivity of each selection method; as selection performance increases the F_1_ score approaches 1 and as selection performance decreases the F_1_ score approaches0. To estimate the statistical significance of the difference between F_1_ scores of different methods, we followed the methodology described in [24]. This method tests the null hypothesis that the results of two techniques do not really differ; thus, the responses produced by one of the techniques could have just as likely come from the other. We therefore shuffled the responses produced by one of the methods (but not the other), re-computed the F_1_ score, and determined the likelihood that this shuffling procedure would create an F_1_ score at least as large as the F_1_ score derived from the original, unshuffled comparison.

### Feature selection methods

Eight common linear selection methods were used. This included Ordinary Least Square (OLS), a method of estimating parameters in linear regression[25], two stepwise (wrapper-based) regression approaches: Forward (FLS) and Backward (BLS) selection with three different criteria[26], and LASSO regression (LASSO)[27]. Forward selection involves starting with a model with zero variables and iteratively adding a new variable; if the variable results in a significant improvement in fit then it is included in the model. Backward selection is conceptually similar, but starts initially with all variables in the model and iteratively removes variables. We used three criteria frequently used both in backward and forward feature selection, namely Mallow’s *C*_*p*_ criteria (BLS *C*_*p*_ and FLS *C*_*p*_ respectively)[28], adjusted *R*^2^ (BLS *R*^2^ and FLS *R*^2^)[29], and Bayesian Information criterion, (BLS *B* and FLS *B*)[30]. Collectively, these selection methods address the problem of over fitting, and account for number of explanatory variables relative to the number of data points in the model. The selected features are features that are included in the best model which is in turn determined by the corresponding criteria.According to Mallow’s *C*_*p*_criteria the best model is the simplest model where the criteria's value is approximately equal to the number of features[28]. When using adjusted *R*^2^ or, the model selected is one that corresponds to the maximum *R*^2^ value[29]. On the contrary, the model with the lowest value of the Bayesian Information is preferred, when Bayesian Information criterionis employed[30].

We also included least absolute shrinkage and selection operator (LASSO) over linear regression, which performs shrinking and variable selection simultaneously. The tuning parameter that controls the shrinking was chosen by 10-fold cross validation performed by built-in cv.gmnet function from R packege "glmnet" [31].

The performance of these linear selection methods was contrasted against three non-linear methods. Three filter-based methods were tested, including distance correlation (DC), Hilbert-Schmidt Information Criterion (HS), and Hoeffding’s test (HT) of independence.Here we included all features that were not statistically independent from target variable at .05 significance level. Further information on each selection method is as follows.

Distance correlation (DC) [32, 33] is a universal approach to check if two variables are related, not necessarily linearly. It equals zero when the two variables are statistically independent, and equals to one if one variable is a linear function of another. To test for independence, we used permutation bootstrap with ≈500 replicates implemented in R package “energy”[34].

Hilbert-Schmidt Information Criterion (HS) is a non-parametric measure of dependence based on the Eigen-spectrum of covariance operators in the reproducing kernel Hilbert spaces[35]. The corresponding mapping of the two variables is a function that equals to zero when variables are independent, and is high, when variables are dependent. To test for independence, we used permutation bootstrap with ≈500 replicates[36], implemented in R package “dHSIC” [37], and Gaussian kernel It is possible to tune the bandwidth parameter of the kernel to better identify different types of the dependencies. For simplicity, we used bandwidth parameter equal to onethroughout this study.

Hoeffding’s test (HT) of independence is a non-parametric population-based test for statistical independence[38]. The test statistic depends on the rank order of the observations, with the P-values approximated by the linear interpolation table in Hollander and Wolfe [39]. We used Hoeffding’s test implementation in R package “Hmisc”[40].

### Synthetic data

To test the ability of feature selection methods to identify relevant variables, we constructed synthetic datasets wherein a set of predictor variables were associated with a target variable, known a priori. The rest of the features within a given dataset were random. This can be described formally with the following. $X^{K}=\{X_{1}^{K},X_{2}^{K}{\ldots X}_{80}^{K}\}$ are random variables and $X^{R}=\{X_{1}^{R},X_{2}^{R},\ldots X_{9}^{R}\}$ are predictors with the known association with the continuous response. The goal is to identify what variables out of the set *X* = *X*^*N*^∪*X*^*R*^ are identified as relevant for *Y* by different feature selection methods.

To do this, a target variable, *y*, was created by generating *N* random numbers from a uniform distribution, *y*~*U*[−15,15]. Sine, cosine and quadratic functions were used to model the relationships between the target and predictor variables. Specifically, we identified the function’s parameters, so that the corresponding predictor variable ranged from zero and *X*_*max*_, where *X*_*max*_, is a whole number either 4 or 7. We then solved the inverse problem of finding corresponding $x_{i}\epsilon X_{i}^{R}$ to each *y*_*i*_, *x*_*i*_ = *f*^−1^(*y*_*i*_). Thus, the functional relationships used were

$$x_{i}^{R}=\left[(p_{1}(\frac{1.7}{\pi }\sin^{-1}\frac{y_{i}}{10}+\pi +a)+(1-p_{1})(-\frac{1.7}{\pi }\sin^{-1}\frac{y_{i}}{10}+\pi -a)+)\frac{x_{max}}{4}+noise\right],\;(\mathrm{simplesines})$$

$$x_{i}^{R}=\left[(p_{2}(\frac{2}{\pi }\cos^{-1}\frac{y_{i}}{10}+a)+(1-p_{2})(\frac{2}{\pi }\cos^{-1}\frac{y_{i}}{10}+\pi -a))\frac{x_{max}}{4}+noise\right],\;(\mathrm{simple}\;\mathrm{cosines})$$

that were rounded to the nearest whole number for discrete independent variables. We also approximated all $x_{i}^{R}<0$ to 0, and $x_{i}^{R}>x_{max}$ to *x*_*max*_. We used the following combinations for {*a*,*x*_*max*_} = [{0.1,7},{0.5,4}] for discrete variables, and {*a*,*x*_*max*_} = {0.8,7} for continuous variables. *p*_1_ and *p*_2_ here are random numbers, *p*_1_*ϵ*{0,1},*p*_2_*ϵ*{0,1}.,.

Wealso used

$$x_{i}^{R}=\left[(p_{3}(\frac{-b+\sqrt{\left(b^{2}-4h(c-y_{i})\right)}}{2*h})+(1-p_{3})(\frac{-b-\sqrt{\left(b^{2}-4h(c-y_{i})\right)}}{2*h}))+noise\right],\;(\mathrm{simple}\;\mathrm{square})$$

that we also rounded to the nearest whole number for discrete independent variables. Again, all negative values were set to zero, and exceeding *x*_*max*_. to *x*_*max*_. The parameters used for the discrete variables were {*h*,*b*,*c*,*x*_*max*_} = [{5,−20,10,4},{−9,23,−5,4},{−9/7,73/7,−10,7}], and the parameters used for the continuous variables were {*h*,*b*,*c*,*x*_*max*_} = [{5,−20,−10,4},{−10,60,−80,4}]. For datasets containing only discrete variables, the continuous variables were rounded to the nearest whole number.

Altogether, we constructed *R* = 9 variables with a known association with a dependent variable. If more than one solution existed, *x*_*i*_ was taken randomly, with equal probability, out of all the outcomes. Finally, we added uniform noise and rounded the resulting value to the closest integer in the corresponding range. We then added *K* = 80 random features. These random features were defined as follows. First, the range was defined such that each variable was between zero and *x*_*max*_, where *x*_*max*_ is a random whole number between four and ten, with equal probabilities. Then, the feature was filled with a random whole number, with equal probability, between zero and *x*_*max*_ for discrete random features, and uniformly distributed $X_{j}^{K}\epsilon [0,x_{max}]$ for continuous random features.

We made two sets of the experiments. In the first set, we constructed only discrete variables, in which each and every *xϵX* was a whole number. In the other set, five variables $X_{1}^{R}\ldots X_{5}^{R}$ were discrete, and four variables $X_{6}^{R}\ldots X_{9}^{R}$ were continuous. Both sets also included 70 discrete random variables, $X^{K}=\{X_{1}^{K},X_{2}^{K}{\ldots X}_{70}^{K}\}$, and 10 continuous random variables, $X^{K}=\{X_{71}^{K},{\ldots X}_{80}^{K}\}$.

Each *X*_*i*_*ϵX* is vector of length *N*. We investigated cases where *N* = 500, 750, 1000, 1250 and *noise* equal to .5 and 1.

Because we have a priori knowledge about whether each feature *X*_*i*_*ϵX* is related to *Y*, we can compare different feature selection methods. Thus, for each combination of *N* and *noise* we generated 200 synthetic datasets, applied a given method, and then investigated whether each feature was or was not identified as important or unimportant correctly. We then computed F_1_scores, and checked whether the difference between F_1_ scores of different methods is significant.

Note here, that we needed nonlinear relationship without clear trend, and selected relationships fulfill this purpose. At the same time, we are unable to mimic all potential relationships with synthetic dataset, so we used representative database to compare linear and non-linear methods on real data.

### Representative data

The goal of our study was to test the selection performance of each method under typical usage conditions, in which researchers would attempt to identify relevant features within in a large dataset. To do this, we used data from two longitudinal studies on aging in USA: Wisconsin Longitudinal Study of Aging database (WLS) [2] and Health and Retirement Study (HRS) [1]. WLS is a long-term study of Wisconsin high school graduates of 1957, whose health has been tracked longitudinally, via multiple-choice surveys and interviews, for over 50 years. HRS is a longitudinal study on a healthy retirement, and aging, with the data collected through interviews and surveys.

#### WLS dataset

We extracted health information along with several life style activities from the WLS[2], as reported in the module *Computer-Assisted Personal Interviewing* (*CAPI*) *2011 wave*, *Mail*: *Internet module and Mail*: *Social and Civic Participation*, available at *wls_pub_13_04*.*sas7bdat*. The target variable, *health change*, was computed as the difference in HUIM3 health index, a rating scale targeted at measuring general health, between 2011 and 2004. In total, this representative dataset contained 3,028 respondents with 52 independent variables apiece. We aimed to assess the performance of each method at identifying factors that are already known to influence health in old age. To that end, we identified six variables as potentially relevant to this health indicator based on prior research individuals[41–43]: education level (equivalent years of regular education attained by 2011, denoted as “*education*”), alcohol use (number of alcohol symptoms, denoted as *“alcohol”*), tobacco use (including number of packs of cigarettes smoked per day, age of last cigarette smoked and number of years of regular smoking, denoted respectively as “tobacco”, “tabacco1” and “tobacco2”), and the respondent’s previous health score in 2004 (HUIM3 health index, denoted as “health”). We excluded 687 respondents who were missing data for any from these previously listed factors. Missing data were imputed with median for all other lifestyle activities; in all cases, this missing/imputed data amounted to less than 15% of the data per activity.

Data analysis was done on this dataset in three steps. First, we tested each method for feature selection on the complete set of data with all 51 independent variables, which represents analysis conditions wherein researchers do not have strong a priori knowledge to manually reduce a dataset. Second, we again tested each method for feature selection but on a smaller subset of data containing the six variables described above (*“alcohol”*, *“education”*,*”tobacco”*, *“tobacco1”*, *“tobacco2”*, *“health*”). This analysis was repeated on a smaller, third dataset that did not include the *“health”* variable. We then compared the performance of each selection method against its own performance on the smaller dataset in order to determine the influence of other variables on the feature selection performance of each method.

#### HRS dataset

The HRS[1] dataset was targeting variables preserving cognitive health in aging. Here we included individuals of about the same age as in the WLS [2]dataset, between 70–74 years old. Respectively, we extracted cognitive health information along with life-activities data from the HRS, as reported in the modules *Preload*, *Physical Health*, *Leave-behind questionnaires*, *and Cognition* of 2014 wave, and health related variables from module *Physical Health* of 2008 wave. The target variable, *cognitive health change* was computed as the difference between the total number of words, correctly remembered by the respondents during the immediate and delayed recalls in 2014 and 2008.

In total, our second representative dataset contained information on 900 respondents characterized by 80 independent variables. Similarly to the WLS[2] case, we aimed to assess performance of each method for identifying factors that have been known to influence cognitive health in old age. Based on prior research, we identified four variables as potentially relevant to this health indicator [44–46]: education level (equivalent years of regular education attained by 2014, denoted as “*education*”), total alcohol use (total number of alcohol consumed per week, denoted as *“alcohol”*), smoking (total number of cigarettes consumed per day, denoted as “*smoking*”), and level of physical activity(“physical activity”). We excluded 682 respondents who had missing data for any from these previously listed factors. We than imputed missing data with medians for all other variables; in all cases, this missing/imputed data amounted to less than 20% of the data per variable.

## Results

### Synthetic data

A central goal of the present study was to assess the performance of the various feature selection methods under different usage conditions, including differences in sampling noise, dataset size, and whether features were discretely or continuously dependent. To determine this, F_1_ scores were computed for each feature selection method, for two variable types (discrete and continuous), two levels of noise and four samples sizes (Table 1 and Table 2). Overall, the selection performance of the non-linear methods was generally unaffected by any of these parameters and ranged from .73 to .91 (mean score: .83). However, the selection performance of the linear methods varied with all these parameters and was much lower overall (mean score: .26). Unsurprisingly, the performance of the linear methods worsened as noise increased and sample sized decreased. Moreover, selection performance also differed unpredictably for discrete or continuous variables.

**Table 1:** F1 scores for synthetic data feature selection when all variables are discrete.

	F1 score, noise = .5						F1 score, noise = 1					
Method	N						N					
	200	300	500	1000	2500	5000	200	300	500	1000	2500	5000
HS	.78	.79	.81	.82	.82	.81	.76	.8	.82	.81	.81	.81
HF	.83	.89	.91	.92	.91	.91	.73	.84	.91	.91	.91	.91
DC	.80	.81	.82	.83	.82	.82	.76	.8	.83	.82	.82	.82
OLS	.09	.12	.18	.3	.52	.58	.08	.11	.16	.26	.46	.56
BLS *C*_*p*_	.15	.17	.21	.31	.51	.58	.13	.15	.18	.28	.45	.57
BLS *R*^2^	.14	.16	.2	.31	.51	.58	.13	.15	.17	.28	.46	.56
BLS *B*	.08	.1	.12	.21	.45	.63	.06	.08	.1	.17	.36	.56
FLS *C*_*p*_	.14	.17	.21	.31	.51	.58	.12	.15	.17	.27	.45	.57
FLS *R*^2^	.14	.16	.2	.31	.51	.58	.13	.14	.17	.28	.46	.56
FLS *B*	.09	.11	.12	.21	.45	.63	.07	.08	.1	.17	.36	.56
LASSO	.18	.18	.19	.21	.22	.22	.17	.18	.19	.20	.21	.21

**Table 2:** F1 scores for synthetic data feature selection when part of the variables is continuous.

	F1 score, noise = .5						F1 score, noise = 1					
Method	N						N					
	200	300	500	1000	2500	5000	200	300	500	1000	2500	5000
HS	.80	.81	.81	.81	.82	.81	.8	.81	.82	.81	.82	.81
HF	.85	.89	.89	.90	.90	.90	.78	.85	.90	.89	.90	.90
DC	.81	.82	.81	.82	.82	.82	.79	.81	.82	.81	.82	.81
OLS	.08	.13	.17	.30	.46	.55	.09	.11	.17	.26	.43	.51
BLS *C*_*p*_	.13	.16	.20	.31	.46	.55	.13	.15	.18	.28	.42	.51
BLS *R*^2^	.14	.16	.20	.31	.46	.54	.14	.15	.18	.28	.42	.51
BLS *B*	.07	.09	.11	.23	.43	.58	.06	.08	.11	.18	.37	.49
FLS *C*_*p*_	.13	.16	.2	.31	.46	.55	.13	.15	.18	.28	.42	.51
FLS *R*^2^	.13	.15	.2	.31	.46	.54	.14	.15	.18	.28	.42	.51
FLS *B*	.08	.09	.11	.23	.43	.58	.07	.09	.11	.18	.37	.49
LASSO	.17	.19	.19	.21	.21	.22	.17	.17	.19	.20	.21	.21

The difference inF1-score performance between the linear and non-linear methods can be further broken down into False Discovery Rate (FDR) and sensitivity.

**Fig 1:**
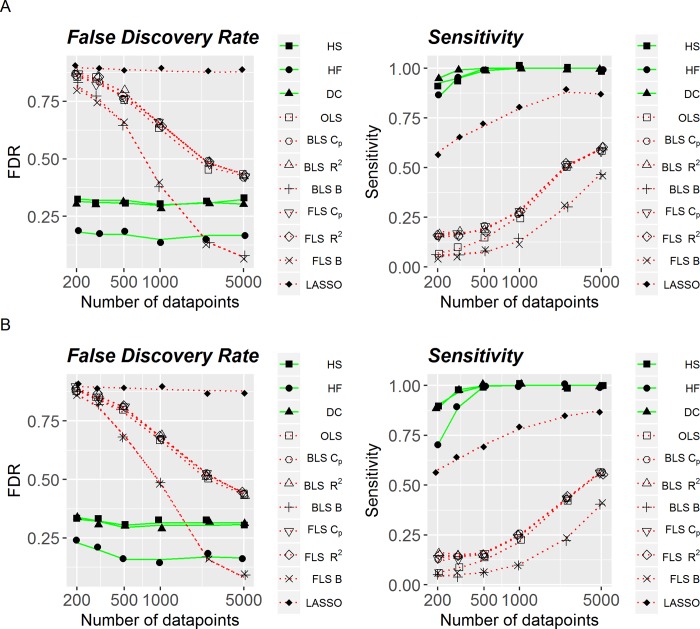
False discovery rate and sensitivity of linear and non-linear methods, with all discrete variables. (A) Added noise is equal to .5. (B) Added noise is equal to 1.

Specifically, Fig 1 is associated with the artificial dataset with all the discrete variables. The right panels show sensitivity as a function of the number of observations (sample size) in two cases: noise parameter is either 0.5 (Fig 1A) or 1.0 (Fig 1B). As can be seen, sensitivity for both linear and non linear methods is increasing with the sample size. However, for any sample size non-linear methods have much higher sensitivity than linear methods, even at larger sample sizes. Among all considered linear methods, LASSO has the highest selection sensitivity at any sample size.

**Fig 2:**
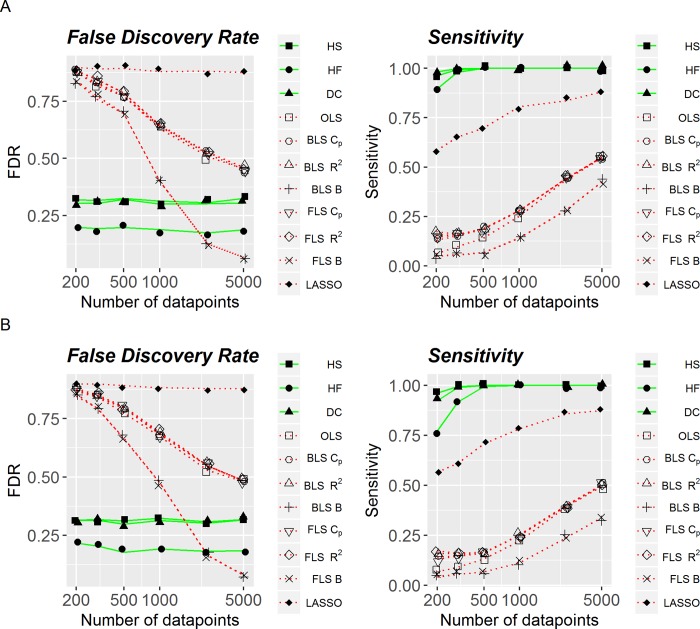
False discovery rate and sensitivity of linear and non-linear methods, with continuous variables. (A) Added noise is equal to .5. (B) Added noise is equal to 1.

Left panels in Fig 1 illustrate FDR as a function of the number of observations. On average, FDR is decreasing with sample size for linear methods, whereas is relatively stable for non-linear ones, ranging around 0.2–0.3. In contrast to sensitivity, performance of LASSO is very poor in the terms of FDR: close to 0.8 regardless of the sample size. In general, FDR tends to be lower for all-non-linear methods under consideration, except for Bayesian Information Criterion when it is used in a situation with very large number of observations (around 5’000), regardless of whether backward or forward feature selection is applied. Similarly to Fig 1, Fig 2 shows accuracy (FDR) and sensitivity as functions of sample size for the artificial data set with continuous variables with the noise parameter equal to 0.5 (Fig 2A) and 1.0 (Fig 2B). Both qualitatively and quantitatively, performance of linear and non-linear methods was very similar to that shown in Fig 1, with non-linear methods being more stable and more optimal across noise levels and different number of observations. Specifically, when sensitivity and accuracy were consolidated into an F1-score, systematic comparisons found that all non-linear methods outperformed all linear methods, *p*< .001.

### Representative data

To further assess the classification performance of each selection approach, we analyzed a subset of the Wisconsin Longitudinal Study of Aging (WLS)[2]and a subset of the Health and Retirement Study (HRS)[1].

#### WLS dataset

Table 3 compares the performance of linear and non-linear methods, showing individual p-values for each of six variables, each being associated with the target variable (changes in health), as suggested by various studies Note that *p* values equal to 1 in the table indicate that the corresponding variable was not included into the final model. The same analysis was performed on three data sets: (i) full dataset with 51 variables; (ii) a subset with only six relevant variables (alcohol, education, three measures on tobacco, health); (iii) a subset with only five relevant variables.

**Table 3:** Comparative performance of non-linear (HS, HF, DC) and linear (BLS *C*_*p*_, FLS *C*_*p*_, BLS *R*^2^, FLS *R*^2^, BLS *BIC*, FLS *BIC*) methods for variable selection, for full set (Panel A), and two subsets (Panels B and C). Shown are *p*-values for associations between health and variables from the Wisconsin Longitudinal Study (WLS)(2).

A. Full set (*51 independent variables*)						
Method	Alcohol	education	tobacco	tobacco1	tobacco2	health
HS	< .01	< .01	< .01	< .01	< .01	< .01
HF	.13	.04	.06	< .01	< .01	< .01
DC	< .01	< .01	< .01	< .01	< .01	< .01
OLS	.02	.39	.35	.44	.83	< .01
BLS *C*_*p*_	.02	1	1	.01	1	< .01
BLS *R*^2^	.03	1	1	< .01	1	< .01
BLS *B*	1	1	1	< .01	1	< .01
FLS *C*_*p*_	.03	1	1	1	< .01	< .01
FLS *R*^2^	.03	1	1	1	< .01	< .01
FLS *B*	1	1	1	1	< .01	< .01
LASSO	.34	.54	.48	.22	.8	.16
B. First subset (*6 independent variables*)						
Method	Alcohol	education	tobacco	tobacco1	tobacco2	health
HS	< .01	< .01	< .01	< .01	< .01	< .01
HF	.13	.04	.06	< .01	< .01	< .01
DC	< .01	< .01	< .01	< .01	< .01	< .01
OLS	.02	. < .01	.56	.92	.36	< .01
BLS *C*_*p*_	.02	< .01	1	1	< .01	< .01
BLS *R*^2^	.02	< .01	1	1	< .01	< .01
BLS *B*	1	< .01	1	1	< .01	< .01
FLS *C*_*p*_	.02	< .011	1	1	< .01	< .01
FLS *R*^2^	.02	< .011	1	1	< .01	< .01
FLS *B*	1	< .01	1	1	< .01	< .01
LASSO	.65	.75	.94	.91	.09	< .01
C. Second subset *(5 independent variables)*						
Method	Alcohol	education	tobacco	tobacco1	tobacco2	
HS	< .01	< .01	< .01	< .01	< .01	
HF	.13	.04	.06	< .01	< .01	
DC	< .01	< .01	< .01	< .01	< .01	
OLS	.057	.06	.10	.58	.74	
BLS *C*_*p*_	.053	.06	.08	.02	1	
BLS *R*^2^	.053	.06	.08	.02	1	
BLS *B*	.04	1	1	1	1	
FLS *C*_*p*_	.06	.07	.12	1	.03	
FLS *R*^2^	.06	.07	.12	1	.03	
FLS *B*	1	1	1	1	.03	
LASSO	.62	.58	.53	.39	.64	

As can be seen in Table 3, selection performance of the three filter-based methods (HS, HF and DC) was unaffected by the number of variables in the dataset. Both Hilbert-Schmidt (HS) and Distance Correlation (DC) identified six variables as significant. Although Hoeffding’s test (HF) only identified four variables as significant, this was still at least as good as the number of variables identified by the linear methods.

By comparison, linear methods for variable selection are sensitive to model misspecification, generating inconsistent results depending on the method and on size of the dataset to be analyzed. Specifically, in the largest dataset, OLS identified as important “*alcohol*”, and “*health*”; BLS *C*_*p*,_, BLS *R*^2^ identified “*alcohol*”, “*tobacco 1*” and “*health*”; BLS *BIC* identified “*tobacco 1*” and “*health*”; FLS *C*_*p*_, and FLS *R*^2^ identified “*alcohol*”, “*tobacco 2*“, and “*health*”, and “*tobacco1*”; and FLS *BIC* identified “*tobacco 2*” and “*health*”. In the smaller dataset, with six relevant variables, OLS identified as important “*alcohol*”, “*education*”, and “*health*”, whereas BLS *C*_*p*,_, BLS *R*^2^, FLS *C*_*p*_, and FLS *R*^2^ identified as important “*alcohol*”, “*education*”, “*tobacco2*” and “*health*”; BLS *BIC* and FLS *BIC* identified “*education*“, “*tobacco 2*” and “*health*”. The exclusion of just one relevant variable, “*health*”, resulted in no variables being selected as important in the smallest dataset by OLS; one variable, “*tobacco 1*” selected by BLS *C*_*p*,_ and BLS *R*^2^; one variable, “*alcohol*”, selected by BLS *BIC*; and only one variable, “*tobacco 2*” selected by all forward selection methods. LASSO was able to select only “*health*” in only one dataset with six variables.

#### HRS dataset

With a small number of relevant variables identified as important, the HRS[1] dataset was used only to compare performance of the linear and non-linear methods of interest, without excluding any variables. The results are given in Table 4, which provides, separately for each method, p-values, each associated with a correlation between *cognitive health change* and one of four relevant variables. Similar to Table 3, a p-value of one indicates that the variable was not included into the final model.

**Table 4:** *P*-values for variables having significant impact on health in the health and retirement study.

Method	alcohol	education	Tobacco	physical activity
HS	.04	.09	.14	< .01
HF	.42	.04	1	.36
DC	.33	.02	.03	.07
OLS	.83	.02	.48	.67
BLS *C*_*p*_	1	< .01	1	1
BLS *R*^2^	1	< .01	1	1
BLS *BIC*	1	1	1	1
FLS *C*_*p*_	1	< .01	1	1
FLS *R*^2^	.26	< .01	1	1
FLS *BIC*	1	1	1	1
LASSO	.53	. < .01	.2	.23

As can be seen, “*education*” was identified by all the methods. In particular, linear methods were able to identify only this variable. In contrast to linear methods, both Hilbert-Schmidt (HS) and Distance Correlation (DC) identified as significant two out of four relevant variables; although Hoeffding’s test only identified one variable as significant, this was again still at least as good as the number of variables identified by the linear methods.

## Discussion

Linear selection methods have been the main methods of the gerontology field to approach and study two of the main central databases, WLS[2] and HRS[1]. In many cases, however, the relationships between variables within these datasets are nonlinear. Although linear methods may still be effective in some cases at identifying important trends in the data, in other cases their selection performance can yield unstable or incorrect results. Because of this, there has been growing interest in the use of non-linear methods for identifying relevant features in aging-related datasets, as these approaches may be better suited for feature selection in many real-world usage scenarios. However, it remains unclear whether these approaches offer superior feature selection performance than linear-based methods, whose operation and implementation are arguably better understood by many researchers. The goal of the present study was to test the effectiveness of linear- and nonlinear-based feature selection methods to identify relevant features marked by non-linear dependencies.

To do this, we assessed seven linear methods (OLS regression; LASSO; forward regression, and backward regression with three different criteria) and three nonlinear methods (distance correlation, Hilbert-Schmidt information criterion and Hoeffding’s test) under both synthetic and real-world use conditions, in which the associations between variables were known in advance. The objective was to assess the ability of each method to select features and discard irrelevant features, and not to assess the accuracy of the various methods at computing the magnitude of the impact of each feature on the dependent variable, such as when computing a coefficient in a linear model. Within the synthetic datasets the variable associations were deliberately non-linear, as we do not usually expect strong linear relationships in real data. Unsurprisingly, selection performance was much better in these cases, with linear methods offering particularly poor detection sensitivity in these cases. There was a small but consistent performance advantage for Hoeffding’s test bases on F1 statistics in all synthetic dataset analyses, although it bears highlighting that sensitivity remained relatively high for all three non-linear methods, and FDR relatively low even as sample size decreased and noise increased. At the same time sensitivity of linear methods consistently remained much lower compared to non-linear methods, with the only exception of LASSO regression that had comparable sensitivity and much higher FDR. This is in line with previous studies that also corroborate this phenomena [47, 48]

Similarly, the selection performance of non-linear methods was more consistent than linear methods in the analysis of real-world health data. Notably, the analyses revealed that the selection performance of the two stepwise regression methods (forward and backward feature selection regression) can vary substantially from one case to another, depending on the variables that are included or excluded from the analysis. Indeed, this was so severe that the removal of just a single variable—in this case, the respondent’s health at the previous assessment—led to substantial differences in the selection of all other features. By comparison, the selection performance of the non-linear algorithms was unaffected by the number of variables in the dataset, which is an important benefit for data-driven analyses in which researchers have weak (or no) a priori hypotheses about the data. Moreover, the selection performance of these non-linear methods was superior to that of linear methods, as evidenced by the number of features identified as significant. Although the poorest-performing method in this analysis, Hoeffding’s test, failed to identify either alcohol consumption or the number of cigarettes smoked per day as significant on health, the number of features it identified as significant (4) was still equal to the best-case selection performance of the linear methods.

We should acknowledge, that our work is limited by its inherent assumptions. First, we only investigated datasets where relationships between target variable and features were non-linear. For further understanding of feature selection method's performance, comparison of different methods applied to datasets were underlying dependencies are linear would be quite beneficial. Second, we explored method's applications when target variable was continuous.In reality, however, target variable is often discrete, i.e. presence or absence of a given disease. Understanding performance of different feature selection methods applied to datasets where target variable is non-continuous will also facilitate better choice of feature selection methods.
